# Mammary Adipose Tissue Control of Breast Cancer Progression: Impact of Obesity and Diabetes

**DOI:** 10.3389/fonc.2020.01554

**Published:** 2020-08-07

**Authors:** Vittoria D’Esposito, Maria Rosaria Ambrosio, Mario Giuliano, Serena Cabaro, Claudia Miele, Francesco Beguinot, Pietro Formisano

**Affiliations:** ^1^URT Genomics of Diabetes, Institute of Experimental Endocrinology and Oncology, National Research Council, Naples, Italy; ^2^Department of Translational Medicine, University of Naples Federico II, Naples, Italy; ^3^Department of Clinical Medicine and Surgery, University of Naples Federico II, Naples, Italy

**Keywords:** mammary adipose tissue, breast cancer, obesity, diabetes, molecular signals, adipocytes, mesenchymal stem cells

## Abstract

Mammary adipose tissue (AT) is necessary for breast epithelium. However, in breast cancer (BC), cell-cell interactions are deregulated as the tumor chronically modifies AT microenvironment. In turn, breast AT evolves to accommodate the tumor, and to participate to its dissemination. Among AT cells, adipocytes and their precursor mesenchymal stem cells (MSCs) play a major role in supporting tumor growth and dissemination. They provide energy supplies and release a plethora of factors involved in cancer aggressiveness. Here, we discuss the main molecular mechanisms underlining the interplay between adipose (adipocytes and MSCs) and BC cells. Following close interactions with BC cells, adipocytes lose lipids and change morphology and secretory patterns. MSCs also play a major role in cancer progression. While bone marrow MSCs are recruited by BC cells and participate in metastatic process, mammary AT-MSCs exert a local action by increasing the release of cytokines, growth factors and extracellular matrix components and become principal actors in cancer progression. Common systemic metabolic diseases, including obesity and diabetes, further modify the interplay between AT and BC. Indeed, metabolic perturbations are accompanied by well-known alterations of AT functions, which might contribute to worsen cancer phenotype. Here, we highlight how metabolic alterations locally affect mammary AT and interfere with the molecular mechanisms of bidirectional communication between adipose and cancer cells.

## Introduction

Breast cancer (BC) is the most common tumor in women and represents the second cause of cancer-caused death after lung cancer (1). In 2018, over 2 million new BC cases were estimated worldwide (2). In the past 3 decades, patient survival rate has increased, thanks to improvements in treatment and detection (3). However, patients’ quality of life is still negatively affected by chemotherapy side effects. Targeted and hormone therapies in most cases do not have long lasting effects; and a number of patients display or acquire resistance to treatments, with a significant reduction of therapy efficacy (3, 4).

The increased incidence and the worse prognosis for BC are parallel to the alarming increase of metabolic disturbances. BC risk is about twofold higher in obese and 16% higher in women with type 2 diabetes (T2D), independently of obesity (5). Patients with obesity and T2D have larger tumors at diagnosis, and worse outcome, with increased risk of distant metastases and mortality (6–8). Moreover, obesity and T2D affect chemotherapy toxicity and surgical complications (9–11).

Breast tissue is composed by 90% of adipose tissue (AT) with permanent interactions between epithelial cells and adipose cells (12). Adipocytes and their precursor mesenchymal stem cells (MSCs) may sustain tumor phenotypes by either acting as energy reservoirs for neighboring cancer cells or through secretion of signaling molecules and vesicles containing proteins, lipids and nucleic acids (13, 14). The dysfunction of AT is now considered a central mechanism for the development of obesity and T2D metabolic complications (15).

In this manuscript, we overview the role of mammary AT as a support for BC cell growth and progression, and describe the known molecular mechanisms underlying the AT/tumor bidirectional crosstalk, especially in the presence of metabolic disorders.

## Mammary Adipose Tissue and Breast Cancer

AT is a loose connective tissue characterized by marked cellular heterogeneity. It is made up of about one-third of adipocytes and two-thirds of stromal-vascular fraction cells, a combination of MSCs, endothelial precursor cells, fibroblasts, smooth muscle cells, pericytes, macrophages and preadipocytes in various stages of development (16). MSCs are located in perivascular niches and participate to cell turnover and to the vascular network for AT tropism (17). For a long time, AT has been considered as an energy depot. Since 90s, the role of AT has been revised and broadened to an active endocrine organ, able to control systemic energy and metabolic homeostasis through a complex network of signals (16, 18).

In the mammary gland, adipose cells are characterized by high plasticity and support the growth and function of the mammary epithelium (19). Mammary AT surrounds the epithelial ducts, which are the milk-producing structures of the breast. *In vitro* and *in vivo* studies have shown the importance of mammary AT for the growth, the branching and the preservation of the ducts and for the functional differentiation of the epithelium ahead of pregnancy (20). For instance, *A-Zip* mice, which have mammary gland lacking mature adipocytes, display rudimental epithelial ducts with reduced branching and severe distention (21).

The role of AT, and more specifically of adipocytes and their precursors MSCs, in BC progression and metastasis, is a quite new area of research. However, adipose cells communicate with cancer cells within the breast, and this may contribute to cancer progression, through different mechanisms.

• *Release of signaling molecules*: Either locally released molecules, either those coming from distal sites converge in the interstitial fluids of the mammary AT (19). Some AT-released factors contribute to AT remodeling, adipogenesis, innervation and angiogenesis by acting through autocrine and paracrine ways. Other AT-factors act in an endocrine manner and influence the functions of many tissues, thus controlling appetite, food intake, glucose disposal and energy expenditure (16, 18, 22). Over 350 proteins have been identified in mammary AT by using proteomic approaches. These factors are called “adipokines” and include leptin, adiponectin, resistin, growth factors (IGF1, insulin-like growth factor 1; VEGF, vascular endothelial growth factor; EGF, epidermal growth factor; FGF, fibroblast growth factor; HGF, hepatocyte growth factor; NGF, nerve growth factor; TGFβ, transforming growth factor), enzymes (autotaxin) and cytokines (interleukin [IL]-1, IL-6, IL-8, CCL5, tumor necrosis factor-TNF-α) (16, 22, 23). These molecules are crucial for the physiology and development of AT and breast epithelium and for the entire organism. However, the same factors may as well contribute to proliferation, motility, invasiveness, epithelial to mesenchymal transition (EMT) and stemness of BC cells, as well as to tumor angiogenesis by activating different molecular mechanisms (12, 24–26).
• *Mechanical support*: Among AT-secreted proteins, there is a wide variety of extracellular matrix (ECM) proteins needed for the tissue structure, but also involved in cell-cell communication systems and in the sequestering of growth factors for a time-and context-dependent release (27). Some of these factors may sustain cancer progression. For instance, adipocyte-derived collagen VI promotes BC progression via bNG2/chondroitin sulfate proteoglycan receptors, while endotrophin, a cleavage product of collagen VI, contributes to tissue fibrosis and EMT of BC cells through enhanced TGF-β signaling (28, 29).
• *Energy supply*: Lipids and metabolites are largely released by AT in mammary glands. Mammary epithelium is able to utilize and metabolize fatty acids to a variety of derivatives (19). However, lipids are taken up also by cancer cells which display a characteristic “lipid metabolic reprogramming.” BC cells take advantage of fatty acids and glycerol for the biosynthesis of membranes, needed for their proliferation, and for the generation of lipid-derived biomolecules such as steroid hormones, diacylglycerol, eicosanoids, phospholipids and sphingolipids which sustain all the functions of cancer cells (12, 30). Recently, it has been shown that the inhibition of fatty acid receptor CD36 impairs metastasis in human BC-derived tumors (31).

Therefore, adipose cells may accommodate BC cells with stimulatory, supportive and nutritive functions.

## BC-Associated Adipose Cells

Emerging evidence indicates that relevant phenotypic changes occur in AT surrounding BC. Indeed, the invasion of AT by BC cells located at the margin of tumor mass is associated with tumor aggressiveness and poor prognosis (32).

Overall, AT adjacent to malignant breast tumors displays down-regulation of the expression of adipogenesis-related genes Homeobox C Cluster (HOXC) 8, HOXC9, fatty acid binding protein 4 (FABP4) and hormone sensitive lipase (HSL) and up-regulation of inflammatory cytokines, like TNF-α and monocyte chemoattractant protein 1 (MCP-1) and of leptin, with a decrease of adiponectin levels (33).

The impact of BC cells specifically on adipocytes has been documented by analyzing adipocytes isolated from human and mouse tumor samples and by using *in vitro* systems of BC cells co-cultured with different types of human and murine adipocytes. Compared to normal adipocytes, the so called “cancer- associated adipocytes” (CAA) are smaller cells, with a reduction in the number and size of lipid droplets and modification of basement membranes and ECM (34). Adipocytes adjacent to breast tumors display increased levels of matrix metalloproteinase-11 (MMP11), which inhibits pre-adipocyte differentiation and reverses mature adipocyte differentiation to maintain the invasive property of cancer cells (34, 35). Impaired adipogenic differentiation program of CAAs is accompanied by the downregulation of Peroxisome Proliferator Activated Receptor Gamma (PPARγ), CCAAT-enhancer-binding protein α (C/EBPα), FABP4, and resisitin mRNA levels (36, 37). Moreover, BC cell-released TNF-α and IL-11 drive a desmoplastic reaction in pre-adipocytes, leading to downregulation of adipogenic master genes (37). PPARγ reduction is also related to the increase of miRNA-155 in exosomes of adipocyte-BC co-coltures (38). Notably, the reduction of lipid droplets takes place with the metabolic reprogramming that adipocytes undergo in contact with BC cells and with the acquisition of a brown-like phenotype. Indeed, cancer cells induce the lipolysis in CAAs *via* HSL and adipose triglyceride lipase (ATGL). Free fatty acids enter into cancer cells, are transported through FABP4 and degraded to provide ATP and bioactive lipids needed for cell invasion, angiogenesis and immunosuppression (11, 30). Consistently, during BC progression, in cancer cells there is an increase of FABP4 and of lipogenic enzymes, like fatty acid synthase (FASN). FASN controls the response of BC cells to E_2_-stimulated ERα signaling and its levels are associated to a poor clinical outcome in patients with BC (39, 40). Gene expression profiling of breast adipocytes shows greater brown adipocyte-related activity next to breast tumors than in benign breast lesions (33). This is consistent with the finding that in co-coltures of adipocytes- BC cells there is an increase of the exosomal miRNA-144 that promotes beige/brown adipocyte differentiation by downregulating MAP3K8/ERK1/2/PPARγ and of exosomal miRNA-126 that plays a crucial role in metabolic reprogramming of adipocytes, targeting IRS1 (Insulin receptor substrate 1) and AMPK (5’ AMP-activated protein kinase) (41). In addition to MMP11, CAAs express MMP1, MMP7, MMP10, MMP14 and PAI, thus damaging ECM integrity in BC environment (34). Furthermore, cancer cells induce adipocytes to secrete fibronectin, which, in turn activates STAT3 signaling pathway in BC cells, thus promoting EMT (42). Fibronectin activates also AKT2 in BC cells, interfering with p38 pathway and docetaxel-induced apoptosis (43). Beside ECM proteins, CAAs display an imbalanced secretion of adipokines, cytokines and growth factors or associated proteins. For instance, compared to normal adipocytes, CAAs secrete a higher amount of leptin, IL-1b, IL-6, CCL5, MCP-1, TNF-α, VEGF and insulin-like growth factor binding protein-2 (IGFBP-2) which in turn promote invasion and metastasis of BC (24, 44, 45). Indeed, CCL5 immuno-detection in peritumoral AT of women with triple negative BC (TNBC) correlates with lymph node and distant metastases and shows a negative correlation with the overall survival of patients (46). In addition, adipocytes co-cultured with BC cells induce the expression of IL-6 in cancer cells, resulting in the phosphorylation of effector kinase CHK1 and the acquisition of a radio-resistant phenotype in BC cells (47). Mature adipocytes also contribute to HER2 + BC cell resistance to trastuzumab-mediated antibody-dependent cellular cytotoxicity and impair immunotherapy efficacy by the hyperexpression of programmed death- ligand 1 (PD-L1), that prevents the antineoplastic functions of CD8 + T cells (24, 48).

Similar to adipocytes, MSCs are largely modified by cancer cells. *Per se* involved in tissue repair, angiogenesis and immunomodulation, MSCs, in contact with cancer cells become cancer supportive cells, so called carcinoma-associated MSCs (CA-MSCs). Nowadays, the contribution of MSCs in BC progression has been investigated mainly by using bone marrow-derived MSCs (BM-MSCs). BM-MSC homing in BC is mediated by tumor (and CAA) –derived chemokines (MCP-1, CCL5, CXCL 16- chemokine [C-X-C motif] ligand 16, SDF1-stromal cell-derived factor 1), growth factors (VEGF, IGF1, TGFβ, FGF) and miRNAs (i.e., miRNA-126/miRNA-126^∗^) (49, 50). Once educated by cancer cells, BM-MSCs secrete CXCL1, CXCL2, SDF1, IL-6, IL-8, TGFβ and microvesicles containing miRNA, such as miRNA-21 and miRNA-34a, all factors implicated in BC survival, progression and chemo-resistance (51, 52). For instance, BC cell-released TNF-α stimulates BM-MSCs to secrete CXCR2 (C-X-C Motif Chemokine Receptor 2) ligands which, in turn, recruit CXCR2 + neutrophils into the tumor, thus promoting metastases (53). Following MSC co-colture, BC cells upregulate IL-6 and CXCL7 pathways with enhanced mammosphere formation efficiency (54). BM-MSCs enhance angiogenesis by releasing soluble factors (VEGF, Leukemia inhibitory factor- LIF, Macrophage Inflammatory Protein 2-MIP2) and exosomes that induce VEGF expression in cancer cells by activating extracellular signal-regulated kinase1/2 (ERK1/2) pathway (55). Moreover, BC cell migration is fostered by BM-MSCs through ER (estrogen receptor)-SDF-1/CXCR4 crosstalk and CXCR2 activation (56, 57). BC cells prompt BM-MSCs to secrete large amount of CCL5, which, in turn, were shown to increase BC metastatic potential of about 5 fold (58). Occurrence of BC metastases in lungs and bones is also supported by the induction of TWIST transcription by BM-MSC production of lysil oxidase enzyme (59) and by the production of exosomes containing miRNA-222/223 (60). Finally, it has been shown that, in contact with BC cells, BM-MSCs are able to transdifferentiate into cancer associated fibroblasts (CAFs), the best companions for BC cells (50, 51, 61).

The crosstalk between BM-MSCs and BC cells is highly relevant since BC typically metastasizes to bone, and bone marrow could represent an ideal environment for the development of BC micro-metastatic niches. However, the interaction of BC cells with mammary AT resident MSCs should be taken into account for its potential role in BC progression.

Recently, it has been shown that MSCs isolated from mammary AT of patients with BC, express high levels of brain-derived neurotrophic factor (BDNF), neurogenic locus notch homolog protein 1 (NOTCH1) and cytoskeletal Vimentin, and reduction of growth differentiation factor 15 (GDF15), IGF1, MMP2, platelet-derived growth factor receptor b (PDGFRB) and TGFβ. Moreover, when co-injected with BC cells in immune-compromised SCID/beige mice, MSCs generate tumors with an increased volume and innervation (52). MSCs isolated from mice bearing BC xenograft tumors can be incorporated in tumor vessels and display up-regulation of SDF1 and α-smooth muscle actin (α-SMA - marker of CAF) (62). Mammary MSCs significantly promote ER-negative BC cell migration and invasion *in vitro* and tumor invasion in a co-transplant xenograft mouse model by producing IL- 6 upon activation of cofilin-1, a well-known regulator of actin dynamics (63). Noteworthy, in another study, using co-culture models, AT-derived MSCs promote BC cell migration and invasion through P2X-mediated purinergic signaling and ATP-loaded microvescicles (64). AT- derived MSC exosomes lead to an up-regulation of WNT target genes Axin2 and Dkk1, and β-catenin in BC cells, thus enhancing cell migration (65). Moreover, mammary MSCs promote mammosphere formation via cytokines, EGF/EGFR/Akt and adipsin pathways (26, 44, 66). In addition, it has been shown that AT-derived MSCs fuse with BC cells spontaneously and this fused population is enriched in BC stem cells (CSC) CD44^+^CD24^–/low^EpCAM^+^ (67). Finally, MSCs isolated from BC patients with pathological stage III disease, induce up-regulation of mRNA expression levels of *IL-4*, *TGF-*β*1*, *IL-10*, *CCR4* and *CD25* in peripheral blood leukocytes and an increase of the percentage of CD4 + CD25(high)Foxp3(+) T regulatory cells *in vitro*, thus sustaining an anti-inflammatory response within the tumor microenvironment (68).

Taken together, these data indicate that both adipocytes and MSCs are largely modified by cancer cells and, once educated by BC cells, become principal actors in metastatic process.

## Adipose Cells as Link Between Metabolic Derangements and BC Progression

Several studies have highlighted that hyperlipidemia, hyperglycemia, hyperinsulinemia and anti-diabetic drugs may be determinant in the association between metabolic imbalance (i.e., obesity and T2D) and BC (7, 8). Changes in body weight and genetic polimorphisms may significantly interact to increase pre- and post-menopausal BC risk (69). However, it is still poorly understood how mammary AT changes related to obesity and T2D conditions might influence BC progression.

In general, in presence of chronic overnutrition, AT expands beyond its capacity in order to maintain a sufficient angiogenesis, leading to persistent hypoxia, fibrosis, cellular senescence, necrotic adipocyte death and a large secretion of pro-inflammatory cytokines, such as TNF-α, IL-6, IL-8 and MCP-1. Compromised AT cells recruit immune cells, particularly monocytes that amplify the local inflammation, determining the “low grade chronic inflammation.” The unhealthy AT expansion largely contributes to the systemic metabolic derangements associated to obesity and T2D (15, 27, 70).

It has been reported that adipocytes and MSCs from human lipoaspirate of obese donors, compared to adipocytes/MSCs from lean subjects, enhance BC cell growth at higher extent and promote tumor metastasis at least in part by IGF1 and leptin pathways (71, 72). Leptin from obese-derived MSCs increases expression of *SERPINE 1*, *SNAI2*, *IL-6*, *TWIST1*, and cyclooxygenase-2 -*COX-2*, which are crucial in EMT and CSC programs in BC cell lines and in TNBC PDX-derived cells (72). Moreover, leptin modulates exosome biogenesis in BC cells and promotes invasive ductal and lobular carcinoma *in vivo* (12, 73). Leptin increase and adiponectin reduction are hallmarks of obesity. While leptin involvement in BC progression is widely recognized (12, 74), the role of adiponectin is still controversial and depends on ERα expression in BC (75). In ERα + cells, low adiponectin levels, like those observed in obesity, stimulate cell proliferation. In contrast, in ERα- cells, adiponectin is able to inhibit cell growth and progression *in vitro* and *in vivo* (76). In AT of obese subjects and mouse models an increase of survivin has also been observed. Survivin is an antiapoptotic protein strongly linked to cancer cell growth. Consistently, its increase in obesity protects MSCs from apoptosis and controls adipocyte lipolysis and lipid storage and may contribute to cancer progression (77, 78). Increased lipid content and external lipid stimuli largely modify adipocyte-BC cell communication. Indeed, lipid-overloaded 3T3-L1 adipocytes transfer about twofold more fatty acids to BC cells, compared to “normal” adipocytes. BC cells in turn up-regulate Carnitine palmitoyltransferase 1A (CPT1A) and electron transport chain complex protein levels and display an increase of proliferation and migration that is parallel to adipocyte lypolisis (79). Mammary adipocytes, incubated with palmitate or oleate or cultured in high glucose medium release higher amounts of IGF1 and CCL5 and promote BC cell proliferation and invasiveness (46, 71). High glucose concentrations also stimulate mammary adipocytes to secrete IL-8, which contributes to tamoxifen resistance in BC cells possibly through up-regulation of Connective Tissue Growth Factor (CTGF) (80). CTGF levels in tumoral tissues of patients with ER + BC correlates with hormone therapy resistance, distant metastases, reduced overall and disease-free survival (80). In addition, tumor-surrounding adipocytes induce multi-drug resistance in BC cells through up-regulation of a transport-associated major vault protein (MVP) which mediates the efflux of the chemotherapeutic agent doxorubicin. This effect is amplified by obesity (81). Recently, it has been shown that in mice bearing triple negative BC, diet-induced obesity (DIO) inhibits fatty acids storage and amplify local inflammation in mammary AT. In parallel, cancer cells increase fatty acid synthesis and change fatty acid composition. Lipid saturated cell membranes protect cancer cells from the cytotoxic effects of doxorubicin (82). Moreover, lipid peroxidases secreted by visceral AT of DIO rats are able to modify carcinogenesis-related genes in non-tumoral breast epithelium cells, thus indicating that in obesity, AT-secreted factors are also involved in early stages of tumor development (83). AT hypoxia and BC progression seem to be strongly linked. A high amount of adipocytes enhances cancer progression inducing the expression of Hypoxia-inducible factor-1α (HIF-1α) and its target genes, which causes the loss of ERα protein in BC cells (84). A positive correlation between breast adipocyte size and the presence of crown-like structures (CLS; spread inflammatory loci characterized by M1 macrophages around necrotic adipocytes) in peritumoral AT has been observed, likely reflecting local inflammation. In patients with both obesity and BC, the presence of CLS accumulation in mammary AT is concomitant with aggressive, high-grade tumors and positive lymph node involvement (85). In the inflamed breast tissue of obese BC patients, higher levels of both NF-κB binding activity and aromatase expression have been reported (85). Mammary AT inflammation, characterized by CLS, inflammatory cytokines and recruitment of a number of immune cells, is largely considered the link between metabolic derangements and worse BC prognosis (70, 86). For this reason, great efforts are being made for disrupting the obesity-cancer link by targeting inflammation with omega-3 fatty acids, non-steroidal anti-inflammatory drugs, monoclonal antibodies against specific cytokines, selective COX-2 inhibitors or polyphenolic compounds. Resveratrol, for example, appears to protect against the protumorigenic effects of obesity in a murine model of BC, at least in part, by inhibiting adipocyte hypertrophy, CLS formation, pro-inflammatory cytokines and COX-2 expression (87).

## Conclusion and Perspectives

**FIGURE 1:**
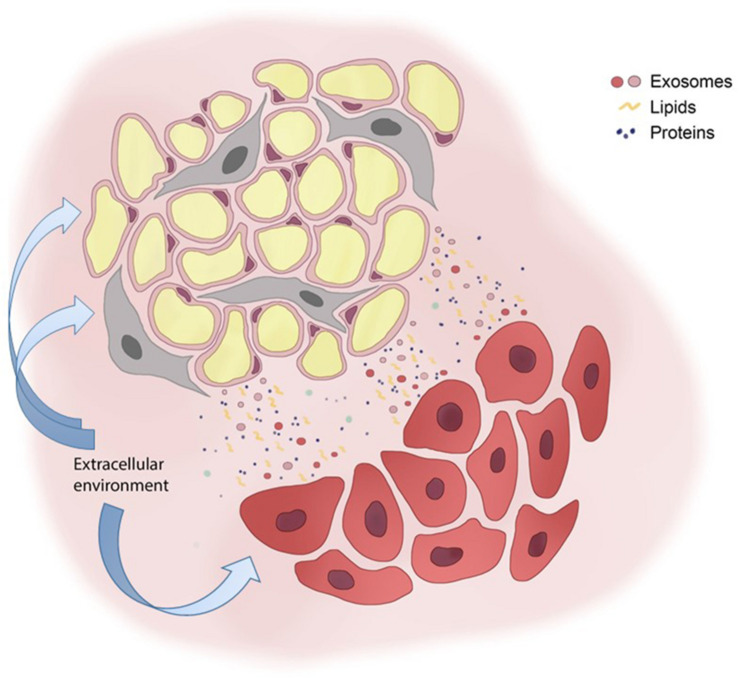
Adipose and breast cancer cell bidirectional communication. Adipocytes (yellow round cells) and mesenchymal stem cells (gray elongated cells) communicate with breast cancer cells (red cells) with a plethora of signals that include exosomes, lipids and proteins. The extracellular environment affects either adipose cells either cancer cells, thus interfering on cell-cell communication systems.

As genetic and epi-genetic mutations accumulate in cancer cells (88, 89), functional alterations appear in AT and support BC progression. AT and BC cells communicate through a network of exosomes, lipids and proteins; extracellular environment may further interfere with these signals (Figure 1). However, most of the studies are based on MSCs/adipocytes isolated from different AT reservoirs (i.e., abdominal, dermal, umbilical), both of murine and human origin. Since the functional diversity of AT depots is well established (20), it is necessary to strengthen the research activity on AT-BC communication in the context of the breast. In addition, the expanded crosstalk of adipocytes and MSCs between each other and with other components of breast microenvironment, such as endothelial cells, pericytes and immune cells should also be exploited concerning BC progression.

## Author Contributions

VD’E, PF, CM, and FB conceived the idea and edited the manuscript. VD’E, MA, and MG wrote the manuscript. SC contributed to literature search and prepared the figure. PF supervised the final version of the manuscript. All authors contributed to the article and approved the submitted version.

## Conflict of Interest

The authors declare that the research was conducted in the absence of any commercial or financial relationships that could be construed as a potential conflict of interest.

## References

[1] GLOBOCAN. (2018). Available online at: https://acsjournals.onlinelibrary.wiley.com/doi/full/10.3322/caac.21492

[2] GLOBOCAN. (2018). Available online at: https://gco.iarc.fr/today/data/factsheets/cancers/20-Breast-fact-sheet.pdf

[3] Miller KD Siegel RL Lin CC Mariotto AB Kramer JL Rowland JH Cancer treatment and survivorship statistics, 2016. *CA Cancer J Clin.* (2016) 66:271–89. 10.3322/caac.21349 27253694

[4] Vasan N Baselga J Hyman DM. A view on drug resistance in cancer. *Nature.* (2019) 575:299–309. 10.1038/s41586-019-1730-1 31723286 PMC8008476

[5] American Cancer Society. *Breast Cancer Facts & Figures 2017–2018.* Atlanta: American Cancer Society, Inc (2017).

[6] Buono G Crispo A Giuliano M De Angelis C Schettini F Forestieri V. Combined effect of obesity and diabetes on early breast cancer outcome: a prospective observational study. *Oncotarget.* (2017) 8:115709–17. 10.18632/oncotarget.22977 29383194 PMC5777806

[7] Gallagher EJ LeRoith D. Obesity and diabetes: the increased risk of cancer and cancer-related mortality. *Physiol Rev.* (2015) 95:727–48. 10.1152/physrev.00030.2014 26084689 PMC4491542

[8] Klil-Drori AJ Azoulay L Pollak MN. Cancer, obesity, diabetes, and antidiabetic drugs: is the fog clearing? *Nat Rev Clin Oncol.* (2017) 14:85–99. 10.1038/nrclinonc.2016.120 27502359

[9] Renehan AG Harvie M Cutress RI Leitzmann M Pischon T Howell S How to manage the obese patient with cancer. *J Clin Oncol.* (2016) 34:4284–94. 27903151 10.1200/JCO.2016.69.1899

[10] Renehan AG Yeh HC Johnson JA Wild SH Gale EA Møller H. Diabetes and cancer research consortium. diabetes and cancer (2): evaluating the impact of diabetes on mortality in patients with cancer. *Diabetologia.* (2012) 55:1619–32. 10.1007/s00125-012-2526-0 22476948

[11] Hoy AJ Balaban S Saunders DN. Adipocyte-tumor cell metabolic crosstalk in breast cancer. *Trends Mol Med.* (2017) 23:381–92. 10.1016/j.molmed.2017.02.009 28330687

[12] Pallegar NK Christian SL. Adipocytes in the tumour microenvironment. *Adv Exp Med Biol.* (2020) 1234:1–13. 10.1007/978-3-030-37184-5_1 32040851

[13] Park J Morley TS Kim M Clegg DJ Scherer PE. Obesity and cancer—mechanisms underlying tumour progression and recurrence. *Nat Rev Endocrinol.* (2014) 10:455–65. 10.1038/nrendo.2014.94 24935119 PMC4374431

[14] Bandini E Rossi T Gallerani G Fabbri F. Adipocytes and microRNAs crosstalk: a key tile in the mosaic of breast cancer microenvironment. *Cancers (Basel).* (2019) 11:E1451. 10.3390/cancers11101451 31569710 PMC6826993

[15] Longo M Zatterale F Naderi J Parrillo L Formisano P Raciti GA Adipose tissue dysfunction as determinant of obesity-associated metabolic complications. *Int J Mol Sci.* (2019) 20:E2358. 10.3390/ijms20092358 31085992 PMC6539070

[16] Frühbeck G. Overview of adipose tissue and its role in obesity and metabolic disorders. *Methods Mol Biol.* (2008) 456:1–22. 10.1007/978-1-59745-245-8_1 18516549

[17] Zuk PA Zhu M Ashjian P De Ugarte DA Huang JI Mizuno H Human adipose tissue is a source of multipotent stem cells. *Mol Biol Cell.* (2002) 13:4279–95. 12475952 10.1091/mbc.E02-02-0105 PMC138633

[18] Poulos SP Hausman DB Hausman GJ. The development and endocrine functions of adipose tissue. *Mol Cell Endocrinol.* (2010) 323:20–34. 10.1016/j.mce.2009.12.011 20025936

[19] Hovey RC Aimo L. Diverse and active roles for adipocytes during mammary gland growth and function. *J Mammary Gland Biol Neoplasia.* (2010) 15:279–90. 10.1007/s10911-010-9187-8 20717712 PMC2941079

[20] Zwick RK Guerrero-Juarez CF Horsley V Plikus MV. Anatomical, physiological, and functional diversity of adipose tissue. *Cell Metab.* (2018) 27:68–83. 10.1016/j.cmet.2017.12.002 29320711 PMC6050204

[21] Couldrey C Moitra J Vinson C Anver M Nagashima K Green J. Adipose tissue: a vital in vivo role in mammary gland development but not differentiation. *Dev Dyn.* (2002) 223:459–68. 11921335 10.1002/dvdy.10065

[22] Wang P Mariman E Renes J Keijer J. The secretory function of adipocytes in the physiology of white adipose tissue. *J Cell Physiol.* (2008) 216:3–13. 10.1002/jcp.21386 18264975

[23] Celis JE Moreira JM Cabezón T Gromov P Friis E Rank F Identification of extracellular and intracellular signaling components of the mammary adipose tissue and its interstitial fluid in high risk breast cancer patients: toward dissecting the molecular circuitry of epithelial-adipocyte stromal cell interactions. *Mol Cell Proteomics.* (2005) 4:492–522. 10.1074/mcp.m500030-mcp200 15695426

[24] Wu Q Li B Li Z Li J Sun S Sun S. Cancer-associated adipocytes: key players in breast cancer progression. *J Hematol Oncol.* (2019) 12:95. 10.1186/s13045-019-0778-6 31500658 PMC6734503

[25] Alberobello AT D’Esposito V Marasco D Doti N Ruvo M Bianco R Selective disruption of insulin-like growth factor-1 (IGF-1) signaling via phosphoinositide-dependent kinase-1 prevents the protective effect of IGF-1 on human cancer cell death. *J Biol Chem.* (2010) 285:6563–72. 10.1074/jbc.M109.097410 20044479 PMC2825452

[26] Goto H Shimono Y Funakoshi Y Imamura Y Toyoda M Kiyota N Adipose-derived stem cells enhance human breast cancer growth and cancer stem cell-like properties through adipsin. *Oncogene.* (2019) 38:767–79. 10.1038/s41388-018-0477-8 30177835

[27] Crewe C An YA Scherer PE. The ominous triad of adipose tissue dysfunction: inflammation, fibrosis, and impaired angiogenesis. *J Clin Invest.* (2017) 127:74–82. 10.1172/JCI88883 28045400 PMC5199684

[28] Iyengar P Espina V Williams TW Lin Y Berry D Jelicks LA Adipocyte-derived collagen VI affects early mammary tumor progression in vivo, demonstrating a critical interaction in the tumor/stroma microenvironment. *J Clin Invest.* (2005) 115:1163–76. 15841211 10.1172/JCI23424 PMC1077173

[29] Park J Scherer PE. Adipocyte-derived endotrophin promotes malignant tumor progression. *J Clin Invest.* (2012) 122:4243–56. 10.1172/JCI63930 23041627 PMC3484450

[30] Beloribi-Djefaflia S Vasseur S Guillaumond F. Lipid metabolic reprogramming in cancer cells. *Oncogenesis.* (2016) 5:e189. 10.1038/oncsis.2015.49 26807644 PMC4728678

[31] Berenguer A Prats N Toll A Hueto JA Bescós C Di Croce L Targeting metastasis-initiating cells through the fatty acid receptor CD36. *Nature.* (2017) 541:41–5. 10.1038/nature20791 27974793

[32] Yamaguchi J Ohtani H Nakamura K Shimokawa I Kanematsu T. Prognostic impact of marginal adipose tissue invasion in ductal carcinoma of the breast. *Am J Clin Pathol.* (2008) 130:382–8. 10.1309/MX6KKA1UNJ1YG8VN 18701411

[33] Wang F Gao S Chen F Fu Z Yin H Lu X Mammary fat of breast cancer: gene expression profiling and functional characterization. *PLoS One.* (2014) 9:e109742. 10.1371/journal.pone.0109742 25291184 PMC4188628

[34] Choi J Cha YJ Koo JS. Adipocyte biology in breast cancer: from silent bystander to active facilitator. *Prog Lipid Res.* (2018) 69:11–20. 10.1016/j.plipres.2017.11.002 29175445

[35] Andarawewa KL Motrescu ER Chenard MP Gansmuller A Stoll I Tomasetto C Stromelysin-3 is a potent negative regulator of adipogenesis participating to cancer cell-adipocyte interaction/crosstalk at the tumor invasive front. *Cancer Res.* (2005) 65:10862–71. 16322233 10.1158/0008-5472.CAN-05-1231

[36] Dirat B Bochet L Dabek M Daviaud D Dauvillier S Majed B Cancer-associated adipocytes exhibit an activated phenotype and contribute to breast cancer invasion. *Cancer Res.* (2011) 71:2455–65. 10.1158/0008-5472.CAN-10-3323 21459803

[37] Meng L Zhou J Sasano H Suzuki T Zeitoun KM Bulun SE. Tumor necrosis factor alpha and interleukin 11 secreted by malignant breast epithelial cells inhibit adipocyte differentiation by selectively down-regulating CCAAT/enhancer binding protein alpha and peroxisome proliferator-activated receptor gamma: mechanism of desmoplastic reaction. *Cancer Res.* (2001) 61:2250–5. 11280794

[38] Wu Q Sun S Li Z Yang Q Li B Zhu S Tumour-originated exosomal miR-155 triggers cancer-associated cachexia to promote tumour progression. *Mol Cancer.* (2018) 17:155. 10.1186/s12943-018-0899-5 30359265 PMC6201501

[39] Guaita-Esteruelas S Gumà J Masana L Borràs J. The peritumoural adipose tissue microenvironment and cancer. The roles of fatty acid binding protein 4 and fatty acid binding protein 5. *Mol Cell Endocrinol.* (2018) 462(Pt B):107–18. 10.1016/j.mce.2017.02.002 28163102

[40] Menendez JA Lupu R. Fatty acid synthase regulates estrogen receptor-α signaling in breast cancer cells. *Oncogenesis.* (2017) 6:e299. 10.1038/oncsis.2017.4 28240737 PMC5337623

[41] Wu Q Li J Li Z Sun S Zhu S Wang L Exosomes from the tumour-adipocyte interplay stimulate beige/brown differentiation and reprogram metabolism in stromal adipocytes to promote tumour progression. *J Exp Clin Cancer Res.* (2019) 38:223. 10.1186/s13046-019-1210-3 31138258 PMC6537177

[42] Balanis N Wendt MK Schiemann BJ Wang Z Schiemann WP Carlin CR. Epithelial to mesenchymal transition promotes breast cancer progression via a fibronectin-dependent STAT3 signaling pathway. *J Biol Chem.* (2013) 288:17954–67. 10.1074/jbc.M113.475277 23653350 PMC3689941

[43] Xing H Cao Y Weng D Tao W Song X Wang W Fibronectin-mediated activation of Akt2 protects human ovarian and breast cancer cells from docetaxel-induced apoptosis via inhibition of the p38 pathway. *Apoptosis.* (2008) 13:213–23. 18158623 10.1007/s10495-007-0158-5

[44] Picon-Ruiz M Pan C Drews-Elger K Jang K Besser AH Zhao D Interactions between adipocytes and breast cancer cells stimulate cytokine production and drive Src/Sox2/miR-302b-mediated malignant progression. *Cancer Res.* (2016) 76:491–504. 10.1158/0008-5472.CAN-15-0927 26744520

[45] Wang C Gao C Meng K Qiao H Wang Y. Human adipocytes stimulate invasion of breast cancer MCF-7 cells by secreting IGFBP-2. *PLoS One.* (2015) 10:e0119348. 10.1371/journal.pone.0119348 25747684 PMC4352027

[46] D’Esposito V Liguoro D Ambrosio MR Collina F Cantile M Spinelli R Adipose microenvironment promotes triple negative breast cancer cell invasiveness and dissemination by producing CCL5. *Oncotarget.* (2016) 7:24495–509. 10.18632/oncotarget.8336 27027351 PMC5029717

[47] Bochet L Meulle A Imbert S Salles B Valet P Muller C. Cancer-associated adipocytes promotes breast tumor radioresistance. *Biochem Biophys Res Commun.* (2011) 411:102–6. 10.1016/j.bbrc.2011.06.101 21712027

[48] Duong MN Cleret A Matera EL Chettab K Mathé D Valsesia-Wittmann S Adipose cells promote resistance of breast cancer cells to trastuzumab-mediated antibody-dependent cellular cytotoxicity. *Breast Cancer Res.* (2015) 17:57. 10.1186/s13058-015-0569-0 25908175 PMC4482271

[49] Hill BS Pelagalli A Passaro N Zannetti A. Tumor-educated mesenchymal stem cells promote pro-metastatic phenotype. *Oncotarget.* (2017) 8:73296–311. 10.18632/oncotarget.20265 29069870 PMC5641213

[50] Hill BS Sarnella A D’Avino G Zannetti A. Recruitment of stromal cells into tumour microenvironment promote the metastatic spread of breast cancer. *Semin Cancer Biol.* (2020) 60:202–13. 10.1016/j.semcancer.2019.07.028 31377307

[51] Raffaghello L Dazzi F. Classification and biology of tumour associated stromal cells. *Immunol Lett.* (2015) 168:175–82. 10.1016/j.imlet.2015.06.01 26145459

[52] Plava J Cihova M Burikova M Bohac M Adamkov M Drahosova S Permanent pro-tumorigenic shift in adipose tissue-derived mesenchymal stromal cells induced by breast malignancy. *Cells* (2020) 9:480. 10.3390/cells9020480 32093026 PMC7072834

[53] Yu PF Huang Y Han YY Lin LY Sun WH Rabson AB TNFα-activated mesenchymal stromal cells promote breast cancer metastasis by recruiting CXCR2(+) neutrophils. *Oncogene.* (2017) 36:482–90. 10.1038/onc.2016.217 27375023 PMC5290040

[54] Liu S Ginestier C Ou SJ Clouthier SG Patel SH Monville F Breast cancer stem cells are regulated by mesenchymal stem cells through cytokine networks. *Cancer Res.* (2011) 71:614–24. 10.1158/0008-5472.CAN-10-0538 21224357 PMC3100554

[55] Zhu W Huang L Li Y Zhang X Gu J Yan Y Exosomes derived from human bone marrow mesenchymal stem cells promote tumor growth in vivo. *Cancer Lett.* (2012) 315:28–37. 10.1016/j.canlet.2011.10.002 22055459

[56] Rhodes LV Antoon JW Muir SE Elliott S Beckman BS Burow ME. Effects of human mesenchymal stem cells on ER-positive human breast carcinoma cells mediated through ER-SDF-1/CXCR4 crosstalk. *Mol Cancer.* (2010) 9:295. 10.1186/1476-4598-9-295 21087507 PMC2998478

[57] Halpern JL Kilbarger A Lynch CC. Mesenchymal stem cells promote mammary cancer cell migration in vitro via the CXCR2 receptor. *Cancer Lett.* (2011) 308:91–9. 10.1016/j.canlet.2011.04.018 21601983 PMC3311035

[58] Karnoub AE Dash AB Vo AP Sullivan A Brooks MW Bell GW Mesenchymal stem cells within tumour stroma promote breast cancer metastasis. *Nature.* (2007) 449:557–63. 17914389 10.1038/nature06188

[59] El-Haibi CP Bell GW Zhang J Collmann AY Wood D Scherber CM Critical role for lysyl oxidase in mesenchymal stem cell-driven breast cancer malignancy. *Proc Natl Acad Sci USA.* (2012) 109:17460–5. 10.1073/pnas.1206653109 23033492 PMC3491529

[60] Bliss SA Sinha G Sandiford OA Williams LM Engelberth DJ Guiro K Mesenchymal stem cell-derived exosomes stimulate cycling quiescence and early breast cancer dormancy in bone marrow. *Cancer Res.* (2016) 76:5832–44. 27569215 10.1158/0008-5472.CAN-16-1092

[61] Atiya H Frisbie L Pressimone C Coffman L. Mesenchymal stem cells in the tumor microenvironment. *Adv Exp Med Biol.* (2020) 1234:31–42. 10.1007/978-3-030-37184-5_3 32040853

[62] Muehlberg FL Song YH Krohn A Pinilla SP Droll LH Leng X Tissue-resident stem cells promote breast cancer growth and metastasis. *Carcinogenesis.* (2009) 30:589–97. 10.1093/carcin/bgp036 19181699

[63] Walter M Liang S Ghosh S Hornsby PJ Li R. Interleukin 6 secreted from adipose stromal cells promotes migration and invasion of breast cancer cells. *Oncogene.* (2009) 28:2745–55. 10.1038/onc.2009.130 19483720 PMC2806057

[64] Maffey A Storini C Diceglie C Martelli C Sironi L Calzarossa C Mesenchymal stem cells from tumor microenvironment favour breast cancer stem cell proliferation, cancerogenic and metastatic potential, via ionotropic purinergic signalling. *Sci Rep.* (2017) 7:13162. 10.1038/s41598-017-13460-7 29030596 PMC5640614

[65] Lin R Wang S Zhao RC. Exosomes from human adipose-derived mesenchymal stem cells promote migration through Wnt signaling pathway in a breast cancer cell model. *Mol Cell Biochem.* (2013) 383:13–20. 10.1007/s11010-013-1746-z 23812844

[66] Yan XL Fu CJ Chen L Qin JH Zeng Q Yuan HF Mesenchymal stem cells from primary breast cancer tissue promote cancer proliferation and enhance mammosphere formation partially via EGF/EGFR/Akt pathway. *Breast Cancer Res Treat.* (2012) 132:153–64. 10.1007/s10549-011-1577-0 21584665

[67] Chan YW So C Yau KL Chiu KC Wang X Chan FL Adipose-derived stem cells and cancer cells fuse to generate cancer stem cell-like cells with increased tumorigenicity. *J Cell Physiol.* (2020). 10.1002/jcp.29574 [Epub ahead of print]. 31994190

[68] Razmkhah M Jaberipour M Erfani N Habibagahi M Talei AR Ghaderi A. Adipose derived stem cells (ASCs) isolated from breast cancer tissue express IL-4, IL-10 and TGF-β1 and upregulate expression of regulatory molecules on T cells: do they protect breast cancer cells from the immune response? *Cell Immunol.* (2011) 266:116–22. 10.1016/j.cellimm.2010.09.005 20970781

[69] Cao S Wei F Zhou J Zhu Z Li W Wu M. The synergistic effect between adult weight changes and CYP24A1 polymorphisms is associated with pre- and postmenopausal breast cancer risk. *Breast Cancer Res Treat.* (2020) 179:499–509. 10.1007/s10549-019-05484-6 31696340

[70] Quail DF Dannenberg AJ. The obese adipose tissue microenvironment in cancer development and progression. *Nat Rev Endocrinol.* (2019) 15:139–54. 10.1038/s41574-018-0126-x 30459447 PMC6374176

[71] D’Esposito V Passaretti F Hammarstedt A Liguoro D Terracciano D Molea G Adipocyte-released insulin-like growth factor-1 is regulated by glucose and fatty acids and controls breast cancer cell growth in vitro. *Diabetologia.* (2012) 55:2811–22. 10.1007/s00125-012-2629-7 22798065 PMC3433668

[72] Sabol RA Bowles AC Côté A Wise R O’Donnell B Matossian MD Leptin produced by obesity-altered adipose stem cells promotes metastasis but not tumorigenesis of triple-negative breast cancer in orthotopic xenograft and patient-derived xenograft models. *Breast Cancer Res.* (2019) 21:67. 10.1186/s13058-019-1153-9 31118047 PMC6530039

[73] Giordano C Gelsomino L Barone I Panza S Augimeri G Bonofiglio D Leptin modulates exosome biogenesis in breast cancer cells: an additional mechanism in cell-to-cell communication. *J Clin Med.* (2019) 8:E1027. 10.3390/jcm8071027 31336913 PMC6678227

[74] Andò S Gelsomino L Panza S Giordano C Bonofiglio D Barone I Obesity, leptin and breast cancer: epidemiological evidence and proposed mechanisms. *Cancers (Basel).* (2019) 11:E62. 10.3390/cancers11010062 30634494 PMC6356310

[75] Andò S Naimo GD Gelsomino L Catalano S Mauro L. Novel insights into adiponectin action in breast cancer: evidence of its mechanistic effects mediated by ERα expression. *Obes Rev.* (2020) 21:e13004. 10.1111/obr.13004 32067339

[76] Mauro L Naimo GD Gelsomino L Malivindi R Bruno L Pellegrino M Uncoupling effects of estrogen receptor α on LKB1/AMPK interaction upon adiponectin exposure in breast cancer. *FASEB J.* (2018) 32:4343–55. 10.1096/fj.201701315R 29513571

[77] Ejarque M Ceperuelo-Mallafré V Serena C Gisela P Yaiza N-Á Margarida T-P Survivin, a key player in cancer progression, increases in obesity and protects adipose tissue stem cells from apoptosis. *Cell Death Dis.* (2017) 8:e2802. 10.1038/cddis.2017.209 28518147 PMC5520726

[78] Ju L Zhang X Deng Y Han J Yang J Chen S Enhanced expression of Survivin has distinct roles in adipocyte homeostasis. *Cell Death Dis.* (2017) 8:e2533. 10.1038/cddis.2016.439 28055005 PMC5386358

[79] Balaban S Shearer RF Lee LS van Geldermalsen M Schreuder M Shtein HC Adipocyte lipolysis links obesity to breast cancer growth: adipocyte-derived fatty acids drive breast cancer cell proliferation and migration. *Cancer Metab.* (2017) 5:1. 10.1186/s40170-016-0163-7 28101337 PMC5237166

[80] Ambrosio MR D’Esposito V Costa V Liguoro D Collina F Cantile M Glucose impairs tamoxifen responsiveness modulating connective tissue growth factor in breast cancer cells. *Oncotarget.* (2017) 8:109000–17. 10.18632/oncotarget.22552 29312586 PMC5752499

[81] Lehuédé C Li X Dauvillier S Vaysse C Franchet C Clement E Adipocytes promote breast cancer resistance to chemotherapy, a process amplified by obesity: role of the major vault protein (MVP). *Breast Cancer Res.* (2019) 21:7. 10.1186/s13058-018-1088-6 30654824 PMC6337862

[82] Mentoor I Nell T Emjedi Z Van Jaarsveld PJ De Jager L Engelbrecht AM. Decreased efficacy of doxorubicin corresponds with modifications in lipid metabolism markers and fatty acid profiles in breast tumors from obese vs. lean mice. *Front Oncol.* (2020) 10:306. 10.3389/fonc.2020.00306 32257945 PMC7089940

[83] Crujeiras AB Cabia B Carreira MC Amil M Cueva J Andrade S Secreted factors derived from obese visceral adipose tissue regulate the expression of breast malignant transformation genes. *Int J Obes (Lond).* (2016) 40:514–23. 10.1038/ijo.2015.208 26443342

[84] Rausch LK Netzer NC Hoegel J Pramsohler S. The linkage between breast cancer, hypoxia, and adipose tissue. *Front Oncol.* (2017) 7:211. 10.3389/fonc.2017.00211 28993797 PMC5622311

[85] Morris PG Hudis CA Giri D Morrow M Falcone DJ Zhou XK Inflammation and increased aromatase expression occur in the breast tissue of obese women with breast cancer. *Cancer Prev Res (Phila).* (2011) 4:1021–9. 10.1158/1940-6207 21622727 PMC3131426

[86] Iyengar NM Zhou XK Gucalp A Morris PG Howe LR Giri DD Systemic correlates of white adipose tissue inflammation in early-stage breast cancer. *Clin Cancer Res.* (2016) 22:2283–9. 10.1158/1078-0432.CCR-15-2239 26712688 PMC4854755

[87] Rossi EL Khatib SA Doerstling SS Bowers LW Pruski M Ford NA Resveratrol inhibits obesity-associated adipose tissue dysfunction and tumor growth in a mouse model of postmenopausal claudin-low breast cancer. *Mol Carcinog.* (2018) 57:393–407. 10.1002/mc.22763 29197120 PMC6053655

[88] Hanahan D Weinberg RA. Hallmarks of cancer: the next generation. *Cell.* (2011) 144:646–74. 10.1016/j.cell.2011.02.013 21376230

[89] D’Esposito V Ambrosio MR Perruolo G Libutti M Formisano P. Gene-Environment Interaction and Cancer. In: Teperino R editor. *Beyond Our Genes.* Cham: Springer (2020).

